# Molecular dynamics simulations on the Tre1 G protein-coupled receptor: exploring the role of the arginine of the NRY motif in Tre1 structure

**DOI:** 10.1186/1472-6807-13-15

**Published:** 2013-09-18

**Authors:** Margaret M Pruitt, Monica H Lamm, Clark R Coffman

**Affiliations:** 1 Department of Genetics, Development and Cell Biology, Iowa State University, Ames, IA 50011, USA; 2 Department of Chemical and Biological Engineering, Iowa State University, Ames, IA 50011, USA

**Keywords:** GPCR, Tre1, Molecular dynamics, Germ cell migration, Salt bridge formation

## Abstract

**Background:**

The arginine of the D/E/NRY motif in Rhodopsin family G protein-coupled receptors (GPCRs) is conserved in 96% of these proteins. In some GPCRs, this arginine in transmembrane 3 can form a salt bridge with an aspartic acid or glutamic acid in transmembrane 6. The *Drosophila melanogaster* GPCR Trapped in endoderm-1 (Tre1) is required for normal primordial germ cell migration. In a mutant form of the protein, Tre1^sctt^, eight amino acids RYILIACH are missing, resulting in a severe disruption of primordial germ cell development. The impact of the loss of these amino acids on Tre1 structure is unknown. Since the missing amino acids in Tre1^sctt^ include the arginine that is part of the D/E/NRY motif in Tre1, molecular dynamics simulations were performed to explore the hypothesis that these amino acids are involved in salt bridge formation and help maintain Tre1 structure.

**Results:**

Structural predictions of wild type Tre1 (Tre1^+^) and Tre1^sctt^ were subjected to over 250 ns of molecular dynamics simulations. The ability of the model systems to form a salt bridge between the arginine of the D/E/NRY motif and an aspartic acid residue in transmembrane 6 was analyzed. The results indicate that a stable salt bridge can form in the Tre1^+^ systems and a weak salt bridge or no salt bridge, using an alternative arginine, is likely in the Tre1^sctt^ systems.

**Conclusions:**

The weak salt bridge or lack of a salt bridge in the Tre1^sctt^ systems could be one possible explanation for the disrupted function of Tre1^sctt^ in primordial germ cell migration. These results provide a framework for studying the importance of the arginine of the D/E/NRY motif in the structure and function of other GPCRs that are involved in cell migration, such as CXCR4 in the mouse, zebrafish, and chicken.

## Background

G protein-coupled receptors (GPCRs) are the largest class of membrane proteins, accounting for 2% of genes in the human genome [1-3]. In general, GPCRs are responsible for modulating signals from the extracellular environment and transducing these stimuli into intracellular signaling cascades and cellular responses. GPCRs are involved in a wide range of cellular processes including cell movement, neurotransmission and olfaction, and can also be involved in disease progression with roles in metastasis, angiogenesis, cell proliferation and inflammation [4,5]. Since GPCRs are involved in maintaining homeostasis as well as disease progression, GPCRs are an important group of proteins to study, informing basic cellular and molecular biology as well as pharmaceutical applications.

Although there are many different genes encoding for GPCRs, all GPCRs share a common structure. GPCRs have seven transmembrane α-helices (TM1-TM7) connected by three intracellular and three extracellular loops. There are five main families of human GPCRs (Rhodopsin, Secretin, Glutamate, Adhesion and Frizzled/Taste2) [1-3], and this classification holds true for GPCRs in other bilateral species [6].

GPCRs are inherently difficult to crystallize due to their transmembrane nature and the fact that individual GPCRs are typically expressed at low levels within cells. GPCRs, like other transmembrane proteins, require a membrane-like environment to remain in a properly folded conformation. The required presence of a membrane makes the overexpression and subsequent purification of GPCRs challenging. The first GPCR crystal structure, bovine rhodopsin, was determined in 2000 [7], with nearly seven years passing before a crystal structure for the second GPCR was published. To date there are 16 GPCRs crystallized, all representing the Rhodopsin family of GPCRs [8]. Additionally, six of these proteins (bovine opsin, bovine rhodopsin, human A_2A_-adenosine receptor, turkey *β*_*1*_-adrenergic receptor, human *β*_*2*_-adrenergic receptor, and rat neurotensin receptor NTSR1) have been crystallized in active-like states [8].

Due to the difficulties of GPCR purification and crystallization, protein structure prediction programs and molecular dynamics (MD) simulations are frequently used to investigate the structures of GPCRs. There are currently three computational techniques available to generate a three-dimensional structural prediction of a protein: homology modeling, threading, and *ab initio* modeling. Homology modeling builds a three-dimensional structure by first identifying an evolutionarily related homologous protein with a known structure to use as a template. The program then aligns the amino acid sequence of the protein of interest to the amino acid sequence of the chosen template and finally builds the model [9-11]. The relatively low number of GPCR crystal structures is a major limitation to homology modeling. A lack of diverse structures means that a majority of GPCRs will still lack a homologous protein to use as a template. It is possible to build a highly accurate model when the template protein and the protein sequence of interest share 50% or more sequence identity [9,10]. However, when the sequence identity is below 30%, the protein structure prediction will likely more closely resemble the template structure than the native structure of the protein [12]. The sequence identity between crystallized GPCRs and other known GPCRs is often below 30% [13]. Due to the prevalence of low sequence identity, it is suggested that both sequence identity and structural information be used when choosing the template protein [13].

Threading, similar to homology modeling, is a template-based approach to structure prediction. The first step in threading is to search for evolutionary relatives to the protein sequence of interest. This is commonly accomplished with Position-Specific Iterative Basic Local Alignment Search Tool (PSI-BLAST) [14]. PSI-BLAST generates a sequence profile, which is used by a secondary structure predictor, like PSIPRED [15], to determine the secondary structure of the protein sequence of interest. Both the secondary structure and the sequence profile from PSI-BLAST are used in a threading algorithm to identify template proteins from the Protein Data Bank that have similar protein folds to the sequence of interest. Templates used in threading may show no evolutionary relationship [11]. The use of multiple templates, creating a chimeric GPCR, has been shown to provide a more accurate model than using a single protein template [13,16,17]. Multiple templates can be used in both homology modeling and threading.

*Ab initio* modeling builds a three-dimensional protein model from sequence information alone, without using a template structure, based upon the assumption that the protein structure will assume the lowest free energy conformation [9]. *Ab initio* modeling can work well for proteins with less than 120 amino acids [11]. Although there are three different ways to build a protein structure prediction, some current modeling programs use a combination of approaches to predict a structure [11]. The accuracy of the final model is linked to the template(s) chosen, and some approaches to generating a protein structural prediction work better on certain proteins or parts of proteins than others [18,19].

With only 16 distinct GPCR proteins crystallized, it can be difficult to find a suitable template(s) to use with the modeling software. Part of this challenge has been alleviated by the availability of web servers specifically designed for modeling GPCRs, such as GPCR-ModSim [20] and GPCR-Iterative Threading ASSEmbly Refinement (GPCR-ITASSER) [21-23]. GPCR-ModSim is a server that allows investigators to model GPCRs using MODELLER [9,20,24] and GPCR-ModSim users have the option of choosing whether to align their GPCR sequence with inactive-like crystallized GPCRs or active-like crystallized GPCRs. GPCR-ModSim aligns the sequence and shows the percent identity with the available templates. The user can then choose which template to use and GPCR-ModSim generates a homology model using MODELLER. Once a homology model is generated, the user has the option of submitting it for MD simulations in a solvated 1-palmitoyl-2-oleoyl-*sn*-glycero-3-phosphocholine lipid bilayer [20].

GPCR-ITASSER is another web server that allows for protein structure prediction [21-23]. GPCR-ITASSER takes the initial GPCR sequence and identifies evolutionary relatives using PSI-BLAST and secondary structures using PSIPRED. The results from PSI-BLAST and PSIPRED are used by the Local Meta-Threading Server (LOMETS) to find potential templates in the Protein Data Bank. Any sequence without a matched template is modeled using an *ab initio* helix-modeling program. Additional restraints to the protein structure are incorporated through the use of the online database GPCRRD (GPCR Research Database), which contains experimental restraints from other GPCR databases and literature [21-23]. The *ab initio* modeling, results from threading, and restraints from the GPCRRD are all used to assemble and build a structural model. This structural model is refined using Fragment-Guided Molecular Dynamics [22] to give the final model.

In this study, the GPCR-ModSim [20] and the GPCR-ITASSER [21] web servers were used to predict protein structures of the GPCR Trapped in endoderm-1 (Tre1). Tre1 is a Rhodopsin family GPCR required for proper *Drosophila melanogaster* primordial germ cell migration [25-27]. In a mutant form of the protein, Tre1^sctt^, primordial germ cell migration is severely disrupted. The primordial germ cells scatter across the posterior half of the embryo rather than populating the two gonads. The molecular lesion in *tre1*^*sctt*^ RNA is a point mutation that results in an in-frame deletion of eight amino acids, RYILIACH [27]. Two of these amino acids (RY) are part of the highly conserved D/E/NRY motif in TM3 of Rhodopsin family GPCRs. The D/E/NRY motif is thought to act as a micro-switch in the activation mechanism of Rhodopsin family GPCRs [3,28], and the arginine is conserved in 96% of Rhodopsin family GPCRs [29]. The arginine of the D/E/NRY motif (R3.50 following Ballesteros-Weinstein nomenclature [30]) can form a salt bridge with TM6 in numerous GPCRs [31-42], and while the exact role of the salt bridge is unknown [43], it is clear that the arginine is very important for Tre1^+^ function [27]. The first position of the D/E/NRY motif is also highly conserved. It is an acidic residue (aspartic acid (D) or glutamic acid (E)) in 86% of GPCRs [29]. In some GPCRs, the aspartic acid or glutamic acid can interact with the neighboring arginine to form an intrahelical salt bridge in addition to the interaction with TM6 [36,44]. Since an acidic residue in the first position of the motif is not present in Tre1 (NRY motif), an intrahelical salt bridge with the arginine does not form. This could make the interhelical salt bridge with TM6 more important.

Protein structure predictions of putative inactive structures of both Tre1^+^ and Tre1^sctt^ were generated with GPCR-ModSim [20] and GPCR-ITASSER [21]. The NAMD simulation package [45] was used to perform MD simulations on both Tre1^+^ and Tre1^sctt^ embedded in a 1-palmitoyl-2-oleoyl-*sn*-glycero-3-phosphoethanolamine (POPE) lipid bilayer, and MD simulations were run on four different model systems for over 250 ns each. The proteins were embedded in a POPE lipid bilayer since phosphoethanolamine is the most abundant phospholipid in Drosophila cell membranes [46]. The NRY motif of Tre1 was studied by examining the possibility of salt bridge formation between the arginine of the NRY motif and an aspartic acid residue of TM6. One of the wild type Tre1 model systems shows potential for a strong salt bridge to form between R134 of the NRY motif and D266 of TM6. The distances between the residues are favorable for salt bridge formation and could indicate that the salt bridge promotes interhelical stabilization of the Tre1 GPCR. The lack of similar interactions in the mutant model systems with an alternative arginine residue as well as *in vivo* data with Tre1^sctt^[27] suggests that the arginine of the NRY motif is important to the function and maintenance of an inactive structure that allows for subsequent activation of the Tre1 GPCR.

## Results

### Protein structure prediction

The amino acid sequences for Tre1^+^ and Tre1^sctt^ were used for protein structure predictions using GPCR-ModSim [20] and GPCR-ITASSER [21]. Both GPCR-ModSim and GPCR-ITASSER are web servers for GPCR protein structure prediction, however the web servers differ in the approach taken to generate a protein structure prediction. The GPCR-ModSim server automates the process of using the homology modeling program MODELLER to model GPCRs [20], while GPCR-ITASSER uses multiple threading programs as well as the GPCR Research Database to predict protein structures [21]. These two web servers were used in this study to generate four independent protein structure predictions, two each for Tre1^+^ and Tre1^sctt^. While MODELLER is an established program that has been used to predict GPCR structures for simulations before, this is the first study to use structural predictions from GPCR-ITASSER.

Tre1^+^ and Tre1^sctt^ were modeled to the seven inactive-like GPCRs on the GPCR-ModSim web server. From the multiple sequence alignment generated, it was clear that any one of the seven available GPCR crystal structures could be used as a template to model Tre1^+^ and Tre1^sctt^. However, GPCR-ModSim allows only one template to be chosen, and for both Tre1^+^ and Tre1^sctt^ the template chosen was squid rhodopsin (PDB ID: 2Z73). Not only did squid rhodopsin show the highest total sequence identity to Tre1^+^ and Tre1^sctt^ (17.4% for Tre1^+^ and 16.7% for Tre1^sctt^) (Table 1), but also squid rhodopsin seemed to be the best choice from earlier work using the web server I-TASSER. The I-TASSER web server uses threading to generate a structural prediction of a protein and allows a user to submit a sequence with or without selecting a template to use [11]. The structural prediction from I-TASSER using Tre1^+^ sequence and no selected template looked most similar to the structural prediction when squid rhodopsin was chosen as a template (data not shown). Therefore, squid rhodopsin was selected as the most appropriate template. Using squid rhodopsin as a template, ten models each for Tre1^+^ and Tre1^sctt^ were generated using MODELLER. The two models chosen for further study using MD simulations were selected based on the lowest Discrete Optimized Protein Energy (DOPE-HR) score [47] and are named mtre1 (Tre1^+^) and msctt (Tre1^sctt^).

**Table 1:** **Sequence alignments in GPCR-ModSim indicate squid rhodopsin has the greatest identity to Tre1**^**+ **^**and Tre1**^**sctt**^

**Template**	**Tre1**^**+**^	**Tre1**^**sctt**^
	**Total % identities**	**Total % identities**
1U19 – bovine rhodopsin	14.3	14.1
2RH1 – human *β*_*2*_-adrenergic receptor	15.1	14.6
2VT4 – turkey *β*_*1*_-adrenergic receptor	14.8	14.3
2Z73 – squid rhodopsin *	17.4	16.7
3EML – human A_2A_-adenosine receptor	14.0	13.5
3ODU – human chemokine receptor 4	16.3	14.8
3PBL – human D_3_ dopamine receptor	16.1	15.9

* Denotes the template chosen for homology modeling.

The second set of independent protein structure predictions for Tre1^+^ and Tre1^sctt^ were built using GPCR-ITASSER [21]. The amino acid sequences for Tre1^+^ and Tre1^sctt^ were submitted to GPCR-ITASSER and used in the local threading server to find template proteins. Both Tre1^+^ and Tre1^sctt^ were modeled to Substance P, human *β*_*2*_-adrenergic receptor, bovine rhodopsin and human A_2A_ adenosine receptor. In addition, Tre1^+^ was modeled to turkey *β*_*1*_-adrenergic receptor and human *β*_*2*_-adrenergic receptor-Gs protein complex, while Tre1^sctt^ was modeled to squid rhodopsin. Even though an active GPCR (human *β*_*2*_-adrenergic receptor-Gs protein complex) was a template for the Tre1^+^ model, it is thought that this Tre1^+^ model reflects an inactive conformation of the protein as the resulting model from GPCR-ITASSER is a consensus of restraints from six templates, all but one being inactive. The two best models, based on GPCR-ITASSER confidence scores, were chosen for further study in MD simulations. The models generated by GPCR-ITASSER are named gtre1 (Tre1^+^) and gsctt (Tre1^sctt^).

**Figure 1:**
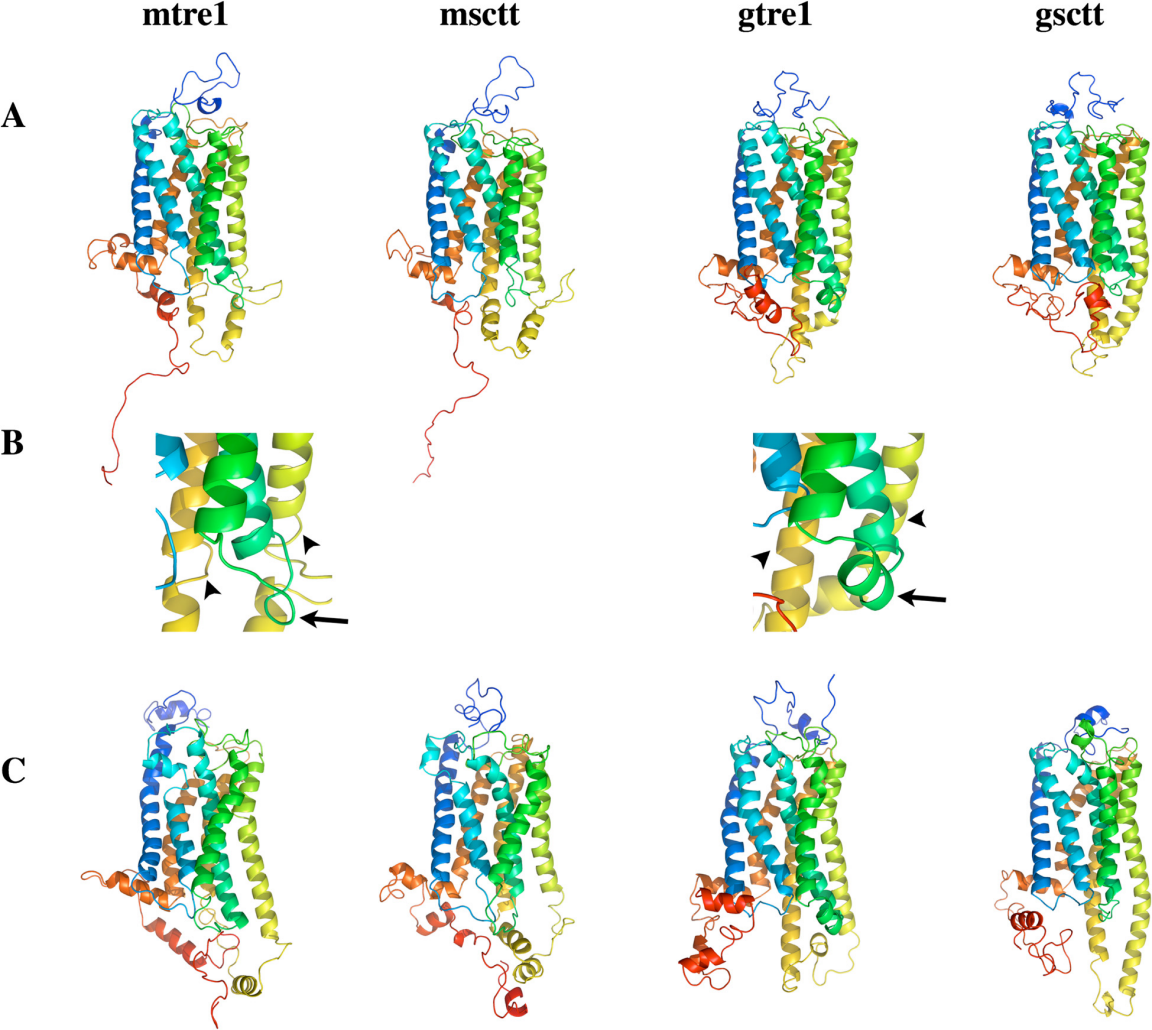
**Three-dimensional models for Tre1**^**+ **^**and Tre1**^**sctt**^**.** mtre1 and msctt are the models generated with GPCR-ModSim and gtre1 and gsctt are the models generated with GPCR-ITASSER. mtre1 and gtre1 are models for Tre1^+^ and msctt and gsctt are models for Tre1^sctt^. The N-termini are colored blue and the C-termini are colored red. **(A)** The best models chosen from the GPCR-ModSim and GPCR-ITASSER. **(B)** A closer view of helices 5 and 6 (yellow and gold) denoted by arrowheads, and intracellular loop 2 (green) shown by arrows. In the gtre1 and gsctt models, helices 5 and 6 are extended compared to the other 5 helices. Intracellular loop 2 is unstructured in mtre1 but contains a short helix in gtre1. **(C)** The resulting models after 262 ns (mtre1), 258 ns (msctt), 270 ns (gtre1) or 276 ns (gsctt) of MD simulations with the protein embedded in a POPE lipid bilayer. The differences in helical length of helices 5 and 6 and the structure of intracellular loop 2 between the initial structures generated by GPCR-ModSim and GPCR-ITASSER are still present after the MD.

The four models, mtre1, msctt, gtre1, and gsctt, selected for further study are shown in Figure 1A. At first glance, all four of the models look similar, but there are distinct differences. Namely, helices 5 and 6 (yellow and gold chains) are roughly the same length as the other five helices in mtre1 and msctt (arrowheads in Figure 1B). In gtre1 and gsctt, helices 5 and 6 are extended relative to the other five helices. Helical extensions of helices 5 and 6 are present in the crystal structure of squid rhodopsin (PDB ID: 2Z73) [48] which was used as a template structure for mtre1, msctt, and gsctt. These differences change the architecture of intracellular loop 3. Additionally, intracellular loop 2 (green) has different structures in mtre1 and gtre1 (arrows in Figure 1B). In mtre1, this loop region is unstructured. In contrast, there is a short helix in intracellular loop 2 in gtre1. These differences in intracellular loop structure can be attributed to the template structure(s) used to generate mtre1 and gtre1. Intracellular loop 2 is of interest since it is the location of some of the residues missing in Tre1^sctt^.

### Building biologically relevant model systems

**Figure 2:**
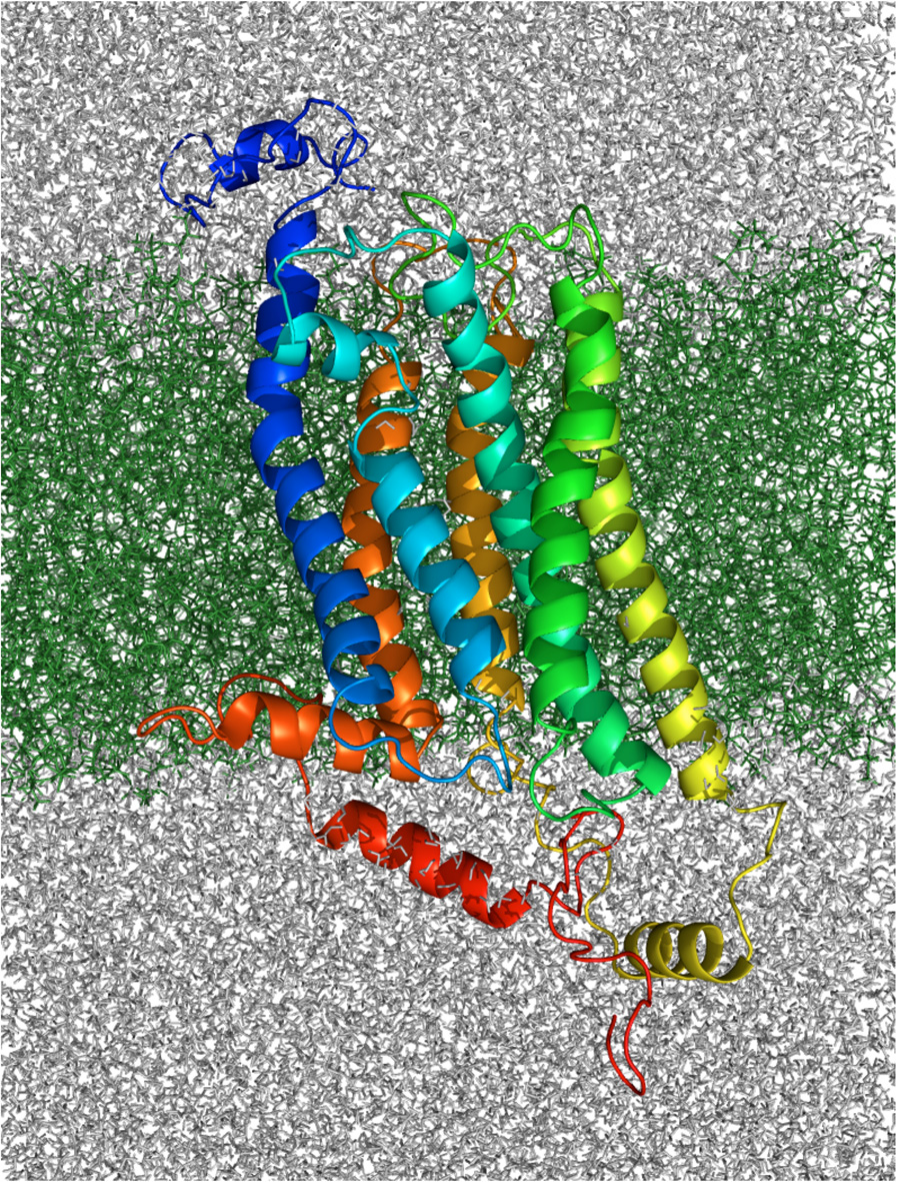
**Protein structure prediction of mtre1 embedded in a POPE lipid bilayer.** The mtre1 model system shown here is after 262 ns of molecular dynamics. The extracellular surface of the bilayer is on top and the intracellular surface of the bilayer is on the bottom. mtre1 is depicted as ribbons, with the N-terminus colored blue and the C-terminus colored red. The POPE bilayer is represented as sticks and is colored green. Water and ions are depicted as grey lines.

As GPCRs exist in a membrane environment, the four different protein structure predictions were inserted into a solvated POPE lipid bilayer using the Membrane Builder in the CHARMM-GUI [49,50]. The final solvated membrane systems are named the same as the structural predictions, mtre1, msctt, gtre1 and gsctt. Each system was subjected to over 250 ns of MD and an example of the mtre1 system after MD is shown in Figure 2.

Experimental work has shown that at 310 K a POPE lipid in a POPE lipid bilayer maintains a surface area of 59.75 – 60.75 Å^2^[51]. To confirm the correct surface areas per lipid were maintained in mtre1, msctt, gtre1 and gsctt, the Voronoi Tesselation and Monte Carlo (VTMC) integration method [52] was used. This method allows for calculation of the surface area per lipid in membrane-lipid systems. VTMC calculates the surface area per boundary and non-boundary lipids. Non-boundary lipids are described as those lipids not interacting with atoms of the protein. It is important to make the distinction between lipid types (boundary versus non-boundary) since lipids interacting with atoms of the protein will have a decreased surface area per lipid. The results of the VTMC analysis are shown in Table 2 and confirm that the non-boundary lipids in each of the model systems maintained the correct surface area per lipid, ranging from 59.0 – 60.2 Å^2^.

**Table 2:** Voronoi Tessellation Monte Carlo integration method confirms the model systems have maintained a fluid-phase bilayer

**Model system**	**Average surface area (Å**^**2**^**/lipid)**	
	**Boundary lipid**	**Non-boundary lipid**
mtre1	45.2	59.8
msctt	45.1	59.0
gtre1	45.4	60.1
gsctt	47.9	60.2

Average surface areas per lipid were calculated using the Voronoi Tessellation Monte Carlo integration method [52] and calculated at the end of each simulation for all model systems. A boundary lipid is a lipid that contacts an atom from the protein and a non-boundary lipid is a lipid that makes no contact with protein atoms. Experimental values of surface area per non-boundary POPE lipid is 59.75 – 60.75 Å^2^[51]. Computationally determined surface area per non-boundary POPE lipid using CHARMM36 is 59.2 Å^2^[66].

### Global movements of the model systems

**Figure 3:**
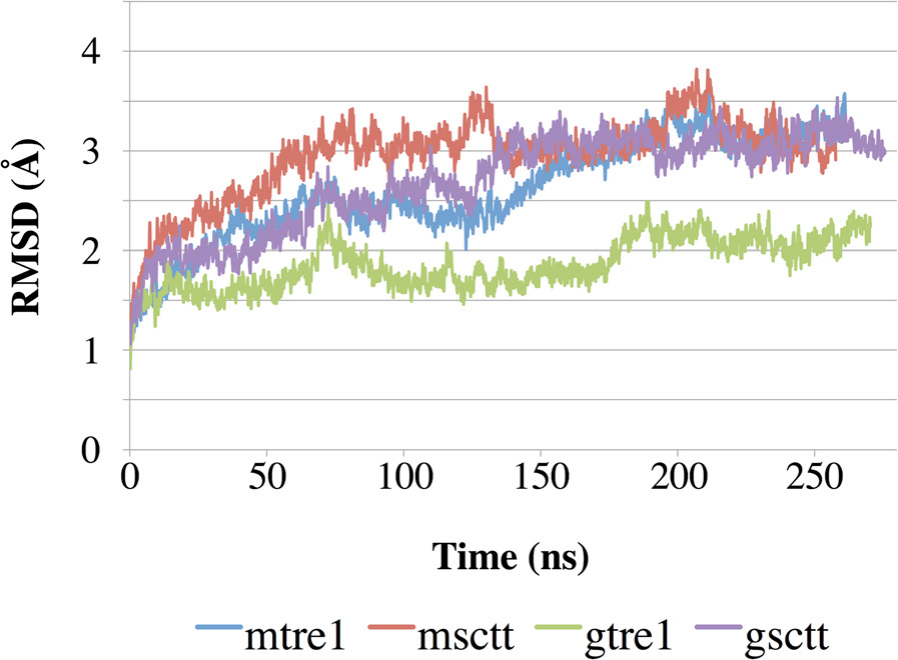
**RMSD of the transmembrane regions shows the protein structures have equilibrated.** Root mean squared deviation (RMSD) was calculated for the Cα atoms of the transmembrane domains and is plotted over simulation time. The transmembrane regions of the protein models do not change much compared to the starting structures over the course of simulations, as the final RMSD values are between 2.0 and 3.5 Å.

Root mean squared deviation (RMSD) was computed over the course of the simulation for the Cα atoms of the transmembrane regions of the proteins to measure structural stability (Figure 3). As seen from the curves in Figure 3, the RMSD values did not change significantly from the starting structure and each curve began to stabilize after 150 ns of dynamics. This suggests that the systems have equilibrated. Further confirmation of structural stability after 150 ns can be seen from the curves of the RMSD values calculated for the complete proteins computed over the course of the simulation (Additional file 1: Figure S1).

**Figure 4:**
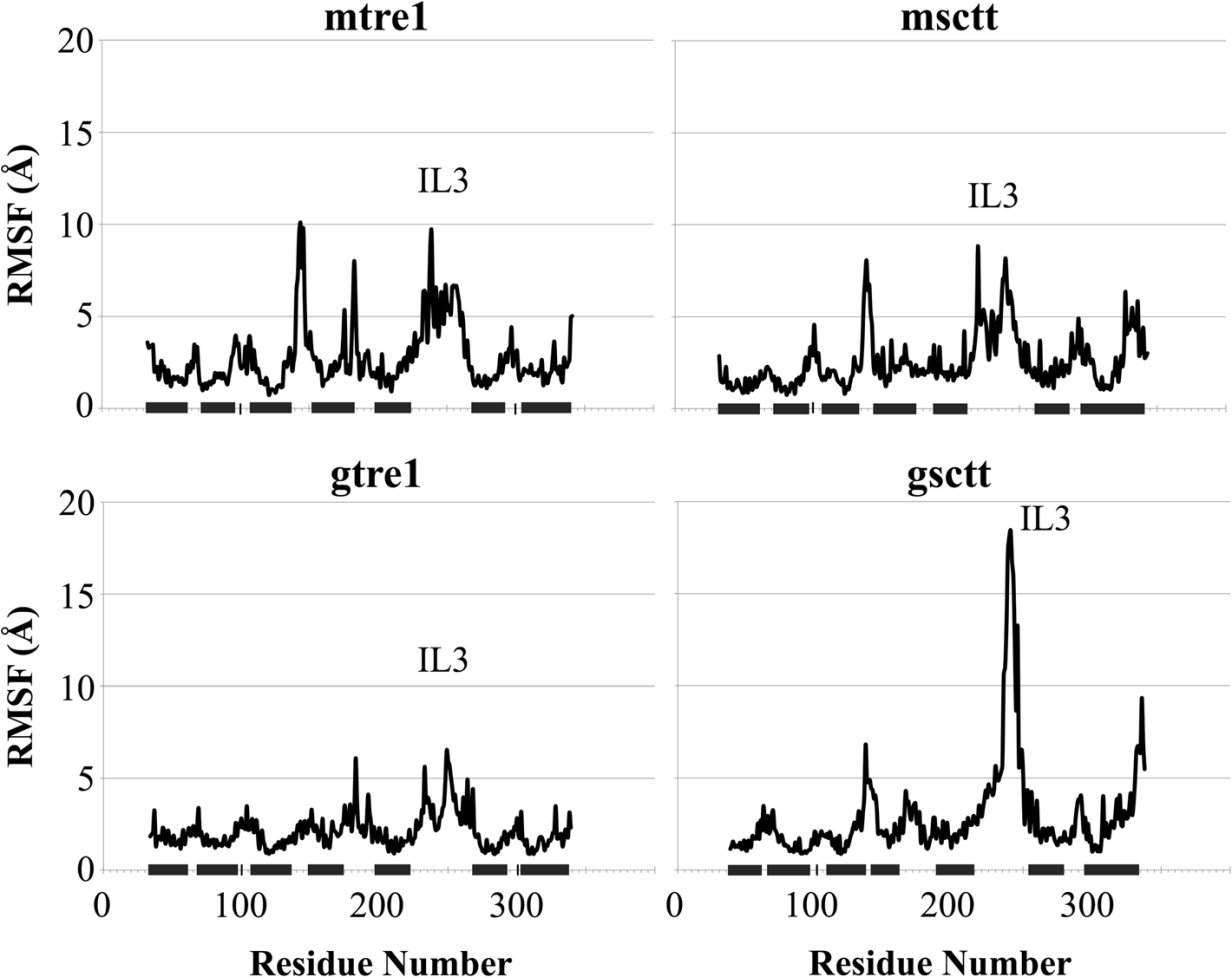
**RMSF of each model system shows transmembrane regions move less than the loop regions.** The root mean squared fluctuation (RMSF) for the mtre1, msctt, gtre1 and gsctt model systems beginning after 150 ns of dynamics are shown. The N- and C-termini are not included in the plots since the termini had high RMSF values and made it difficult to see the fluctuations in the other regions of the protein. The black bars denote the regions of the protein that are within the lipid bilayer. In most model systems, intracellular loop 3 (IL3) shows the greatest fluctuations.

Root mean squared fluctuation (RMSF) was also used as a way to qualitatively characterize the protein dynamics. Here, RMSF describes the fluctuations of each Cα atom of the amino acid residues in the proteins averaged over the simulation time, beginning at 150 ns (Figure 4). The general fluctuations of specific regions of the proteins are similar between each of the Tre1^+^ and Tre1^sctt^ models. It is clear from all four plots that the regions of the protein with the least amount of movement are the transmembrane regions. Intracellular loop 3 shows the greatest fluctuations, which is expected since it is the longest loop in Tre1. Of all the model systems, intracellular loop 3 of gsctt has the highest RMSF values. The high degree of fluctuation seen in intracellular loop 3 of gsctt could be due to its architecture, as intracellular loop 3 is more helical in gsctt than it is in mtre1, msctt, and gtre1. In gsctt, intracellular loop 3 begins as a helical extension of TM5, and then it has an unstructured loop region, followed by another helical region that connects with TM6. The high RMSF values in intracellular loop 3 in gsctt come from the residues in intracellular loop 3 that are in the unstructured loop. It is possible that the more rigid α-helical segments in intracellular loop 3 in gsctt prevent some of the other, unstructured residues in the loop from making important contacts with other parts of the protein. This could cause higher RMSF values.

**Figure 5:**
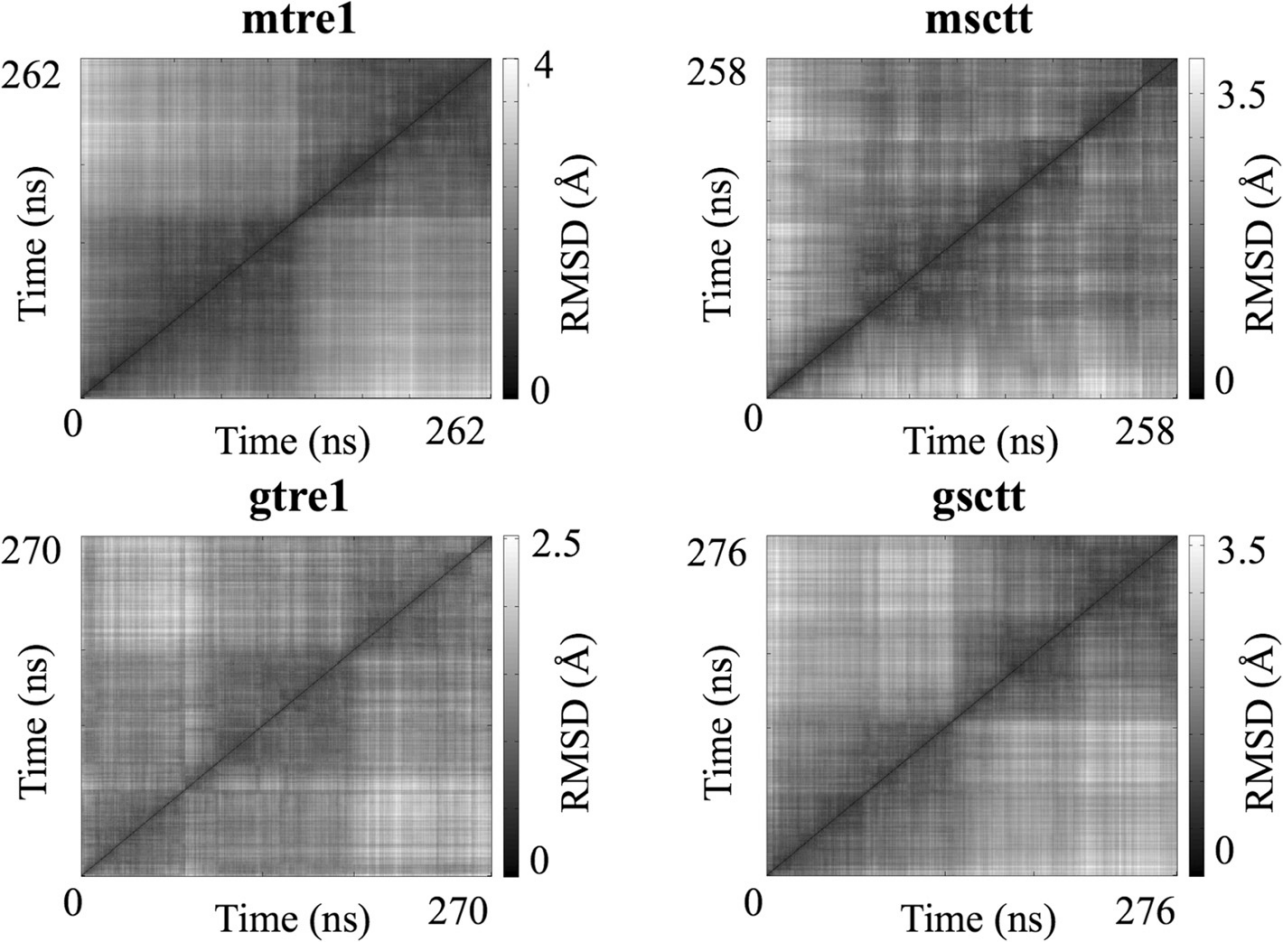
**Heat maps of all-to-all RMSD calculations show sampling of conformational substates during MD simulations.** Transmembrane Cα atoms in mtre1, msctt, gtre1 and gsctt model systems were used in these calculations. The all-to-all RMSD calculation computes the RMSD of all pairs of frames in a trajectory. This all-to-all RMSD calculation was performed using the VMD plug-in RMSD Trajectory Tool [71]. The RMSD scale is different for each model system and is located to the right of each plot.

A third qualitative assessment of the simulations was an all-to-all RMSD calculation on the transmembrane Cα atoms of the Tre1 protein, shown in Figure 5 as heat maps. Heat maps of all-to-all RMSD calculations show the number of different states the protein has visited during the course of the simulation. The darker diagonal blocks in each plot show when the Tre1 protein (Tre1^+^ or Tre1^sctt^) explores conformations that are structurally very similar. Darker off-diagonal blocks suggest that the protein revisits a conformation over the course of the simulation, although in this case, low RMSD alone is not sufficient to guarantee that two, noncontiguous in time, structures are similar [53]. In the heat maps shown here, each simulation samples two or more conformational substates. The quality of sampling is comparable to the sampling obtained at 250 ns in previous MD simulations of GPCRs [53-55].

The final protein structures after the dynamics in a lipid bilayer are shown in Figure 1C (the lipids, water and ions are not shown). In general, the transmembrane regions of the protein structures did not change drastically from the initial structures. Some of the loops have changed considerably, but the distinctions between the initial structures generated by GPCR-ModSim and GPCR-ITASSER noted previously are still present. To quantitatively determine changes in secondary structure over the course of the simulations, the Dictionary of Secondary Structure of Proteins [56,57] was used (Additional file 2: Figure S2). These plots confirm the seven transmembrane helices remain stable throughout the simulation, and the termini and loops are regions of change. While the significance of the differences between the GPCR-ModSim and GPCR-ITASSER models remain to be understood, it is interesting that even after over 250 ns of MD, some of the structural differences between the model systems were not resolved. This could mean that the structural models generated by the different modeling programs represent different protein conformations of Tre1.

### Studies of the NRY motif of Tre1

**Figure 6:**
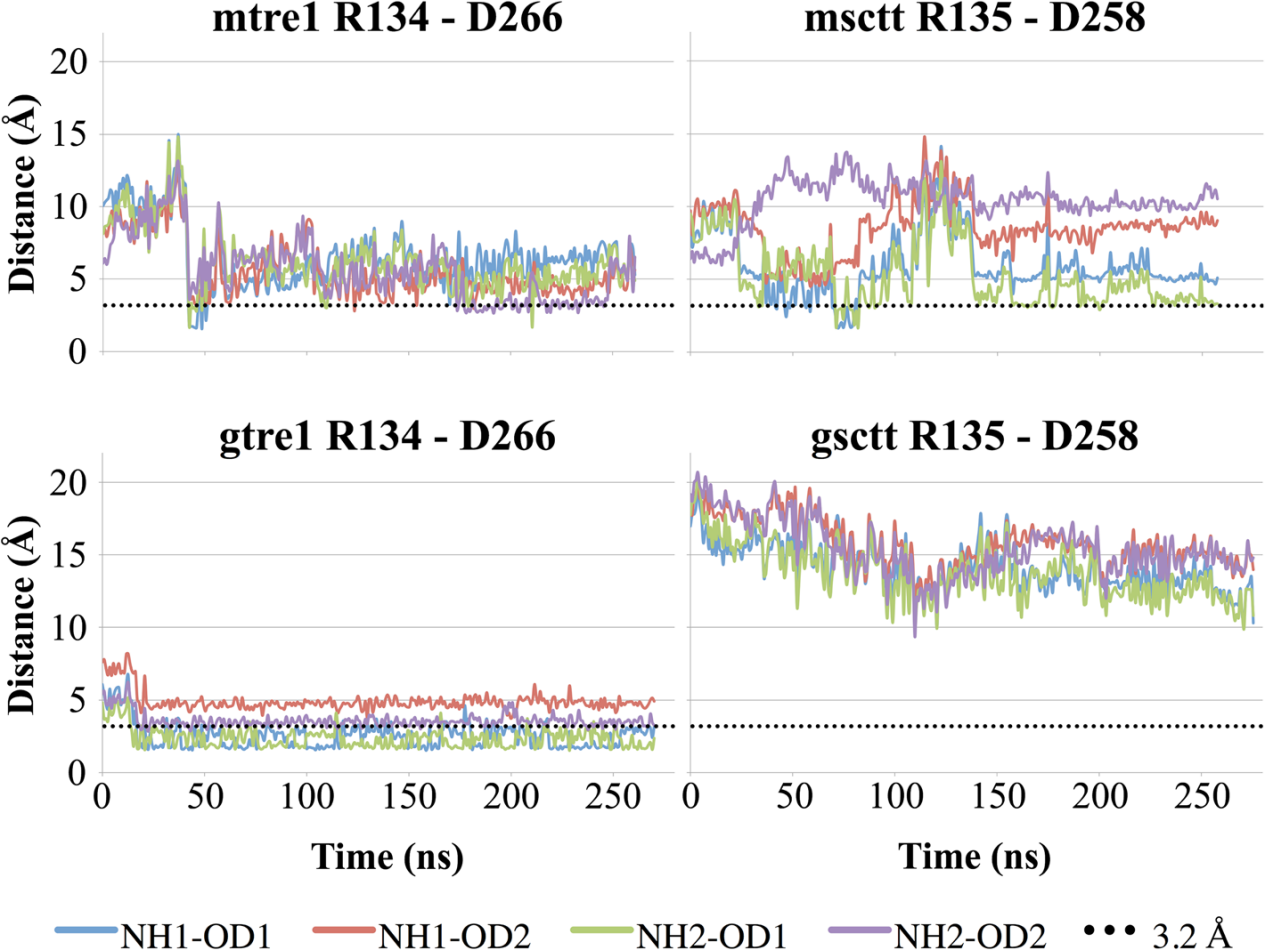
**Distance calculations suggest a salt bridge could form in mtre1, msctt and gtre1 model systems.** Distances between oxygen atoms of the carboxylate group of aspartic acid (D) (OD1, OD2) and nitrogen atoms of the guanidium group of arginine (R) (NH1, NH2) are plotted. N-O distances of 3.2 Å (dotted lines) or less are capable of forming a salt bridge. The N-O distances are close enough to form a salt bridge transiently in mtre1 and msctt. There is potential for a salt bridge to be consistently present in the gtre1 model system. The N-O distances are too great in gsctt to form a salt bridge at any point during the simulation.

**Figure 7:**
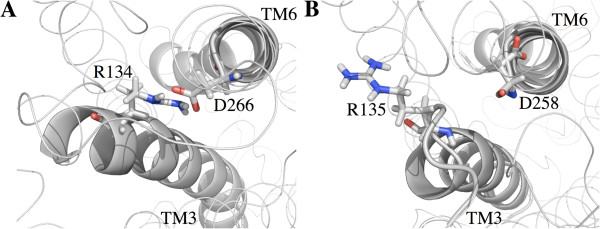
**Orientation of the arginine in gsctt is not correct for salt bridge formation.** Intracellular views of TM3 and TM6 are shown. The arginine and aspartic acid residues studied for formation of a salt bridge are shown as sticks, with the nitrogen atoms labeled blue and the oxygen atoms labeled red. TM3 and TM6 helices are represented as gray ribbons. **(A)** The gtre1 model system at 270 ns. In gtre1, R134 and D266 are close enough to form a salt bridge. Nitrogen – oxygen distances range from 2.4 – 4.9 Å. **(B)** The gsctt model system at 276 ns. The nitrogen atoms of R135 and the oxygen atoms of D266 are not facing each other, which prevents a salt bridge from forming between these residues. Nitrogen – oxygen distances range from 10.3 – 14.8 Å.

From previous genetic studies, it is known that the arginine of the NRY motif (R3.50 following Ballesteros-Weinstein nomenclature [30]) in Tre1 is critical to the function of this GPCR [27]. Other than the critical nature of the arginine to Tre1 function, very little is known about the potential structural roles for this amino acid. It is possible that the arginine of the NRY motif in TM3 is involved in forming a salt bridge with an aspartic acid residue in TM6. A similar salt bridge in other GPCRs is thought to be important for holding GPCRs in inactive or activated states [32-42]. If there is a salt bridge in Tre1 between the arginine of the NRY motif and an aspartic acid residue in TM6, loss of this arginine could remove this salt bridge and impair function, which would be consistent with experimental observations that germ cell migration requires this arginine in Tre1 for function [27]. It is also possible that an alternative arginine just downstream of the deleted amino acids in Tre1^sctt^ could be used to form a salt bridge with TM6. This alternative salt bridge could explain why the *tre1*^*sctt*^ allele does not appear to be a complete loss-of-function allele of the *tre1* gene. In Tre1^+^ the sequence around the arginine is NRYILIACHSR^*^Y. In Tre1^sctt^, the amino acids RYILIACH are missing and the remaining sequence is NSR^*^Y. The arginine of the NRY motif in Tre1^+^ is numbered as R134. The arginine R^*^ in Tre1^sctt^, the alternative arginine, is numbered as R135, meaning this alternative arginine is located one residue from where the original arginine is located in Tre1^+^. Therefore, this alternative arginine could be close enough to form a salt bridge in the Tre1^sctt^ protein. To test this hypothesis, the potential for salt bridge formation was evaluated in all model systems. Here, a salt bridge is defined as a noncovalent interaction between the carboxylate group of aspartic acid (D266 in Tre1^+^ and D258 in Tre1^sctt^) and the guanidium group of arginine (R134 in Tre1^+^ and R135 in Tre1^sctt^). As aspartic acid residues have two oxygen atoms that could be involved in a salt bridge, and arginine residues have two nitrogen atoms that could be involved in a salt bridge, the distance between both of the oxygen atoms of the aspartic acid in TM6 and both of the nitrogen atoms of the arginine in TM3 were calculated and plotted over the simulation time (Figure 6). Interatomic distances of 3.2 Å or less were considered favorable for salt bridge formation and such distances were seen in mtre1, gtre1, and msctt. The atoms studied in gsctt were never close enough to form a salt bridge. As shown in Figure 7B, the nitrogen atoms of R135 in gsctt are not oriented towards the oxygen atoms of the aspartic acid residue in TM6 as they are in the gtre1 system (Figure 7A). The MD simulation of gtre1 shows interatomic N-O distances of 3.2 Å or less for 3 of the 4 possible N-O pairs throughout most of the simulation (NH1-OD1, NH2-OD1, NH2-OD2). N-O distances in mtre1 were consistently greater than 3.2 Å except for ~75 ns towards the end of the simulation (NH2-OD2). The differences in interatomic N-O distances between mtre1 and gtre1 could be due to different conformations of Tre1 being represented or due to inherent differences in the initial protein structure predictions. msctt does not appear to be able to form a stable salt bridge using the alternative arginine, R135, as correct interatomic N-O distances are only transiently seen over the course of the simulation. To ensure the differences in interatomic N-O distances were not due to differences in the distance between TM3 and TM6 carbon backbones, the Cα-Cα distances were measured between the arginine (R134 or R135) in TM3 and aspartic acid (D266 or D258) in TM6 in all systems. Plots of the Cα-Cα distances over the course of the simulation showed that the distances between the residues studied here were similar (Additional file 3: Figure S3).

## Discussion

The Tre1 GPCR is an important component of primordial germ cell migration in Drosophila [25-27]. In a severe partial loss-of-function allele of the *tre1* gene, *tre1*^*sctt*^, proper primordial germ cell migration is disrupted. The Tre1^sctt^ protein is missing eight amino acids, RYILIACH, from the junction of the third transmembrane domain and second intracellular loop [27]. This study was performed to analyze how the loss of the amino acids RYILIACH may affect Tre1 structure.

Protein structure predictions were generated for Tre1^+^ and Tre1^sctt^ using GPCR-ModSim [20] and GPCR-ITASSER [21]. The four resulting structures were inserted into a POPE lipid bilayer and subjected to over 250 ns of MD simulations each. Interesting insights into the structures of Tre1^+^ and Tre1^sctt^ were gained from this study. First, as shown by the RMSD versus time plots and the RMSF plots (Figures 3 and 4), Tre1^+^ and Tre1^sctt^ behave similarly. The RMSD values for both Tre1^+^ and Tre1^sctt^ protein structure predictions begin to stabilize around 150 ns of each MD run. Also, as shown by Figure 4, the general fluctuations of specific regions of the proteins are similar between Tre1^+^ and Tre1^sctt^.

The only primary sequence difference between Tre1^+^ and Tre1^sctt^ is that Tre1^sctt^ is missing the eight amino acids RYILIACH. Of the eight amino acids, the arginine is the most conserved residue, being present in 96% of Rhodopsin family GPCRs [29]. The arginine and the tyrosine are part of a highly conserved D/E/NRY motif in Rhodopsin family GPCRs [3,28]. The D/E/NRY motif is thought to have roles as a micro-switch, being involved in holding the GPCR in an active or inactive state by forming a salt bridge with an aspartic acid residue or a glutamic acid residue in TM6 [31-42]. Interestingly, the arginine is also the most critical residue of the amino acids RYILIACH for proper primordial germ cell migration in Drosophila. When the arginine is substituted by an alanine, a severe loss-of-function germ cell phenotype is observed [27]. The germ cell phenotype from the arginine substitution is indistinguishable from the phenotype when the amino acids RYILIACH are missing. Based upon this knowledge, it was hypothesized that a salt bridge involving the conserved D/E/NRY motif is present in Tre1^+^ and absent in Tre1^sctt^. A salt bridge with the D/E/NRY motif could be important for maintaining Tre1^+^ in a conformation required for efficient ligand binding. The lack of salt bridge in Tre1^sctt^ could alter the protein conformation such that the ligand cannot recognize the receptor. It is also possible that an alternative salt bridge using a nearby arginine could be formed in Tre1^sctt^. If an alternative salt bridge forms, it could be involved in restoring some function of the GPCR.

The ability of the four model systems to form a salt bridge (Figure 6) was examined. Wild-type systems, mtre1 and gtre1, confirm that it is possible for a salt bridge to form between the arginine of the NRY motif (R134) and an aspartic acid (D266) in TM6. The salt bridge analysis using the mutant systems, msctt and gsctt, present a different picture. While it is possible for a salt bridge to form between the alternative arginine (R135) and the aspartic acid of TM6 (D258) in the msctt system, the salt bridge would not be very stable. Distances favorable for salt bridge formation were not consistently present during the simulation (Figure 6). It is clear from Figure 6 that no salt bridge would be expected to form in gsctt. It is possible that the salt bridge seen in gtre1 promotes interhelical stabilization of the protein, and this stabilization could be important for proper function of Tre1. The inability to form a stable salt bridge could disrupt Tre1^sctt^ protein structure making it unable to properly receive its ligand, or could alter the confirmation of Tre1^sctt^ such that it cannot bind interacting proteins. An alternative explanation for the salt bridge analysis results is that the systems have not been sufficiently sampled (Additional file 4: Table S1 and Additional file 5: Table S2), and a salt bridge could still form in the Tre1^sctt^ forms of the protein.

With the salt bridge analysis there are significant differences between the independent model systems for Tre1^+^ and Tre1^sctt^. While it is possible that the different model systems represent two different protein conformations of Tre1, it is also possible that these differences can be attributed to how the protein structure predictions were generated. mtre1 and msctt were built using GPCR-ModSim [20] which uses the homology modeling program MODELLER. gtre1 and gsctt were built using GPCR-ITASSER [21-23] which predicts protein structures through the use of threading. To further study Tre1 structure, a third independent structure of Tre1 could be built. For example, GPCR-Sequence Structure Feature Extractor (GPCR-SSFE) could be used to generate another starting structure. GPCR-SSFE is a database in addition to a homology modeling program that creates homology models of GPCRs using multiple templates and the program MODELLER [24,58]. The ability to use multiple templates is significant since the use of multiple templates with MODELLER has been shown to give more accurate homology models than using a single template [17].

## Conclusions

In this study, the role of the arginine of the NRY motif in Tre1 was investigated. It is known from previous work that this arginine is critical to the proper function of the Tre1 GPCR in Drosophila primordial germ cell migration [27]. Whether or not it is important for Tre1 structure was unknown. The results presented here suggest that a salt bridge may form between this critical arginine and an aspartic acid in TM6 in Tre1.

GPCRs are a common class of proteins involved in cell migration. Similar to how Tre1 is involved in Drosophila primordial germ cell migration, another GPCR, CXCR4, is important for proper primordial germ cell migration in mouse, zebrafish and chicken [59-62]. Like Tre1, CXCR4 contains the highly conserved D/E/NRY motif. While a salt bridge with the arginine of the DRY motif was not present in the crystal structure of the human CXCR4 [63], it would be interesting to learn if the arginine of the DRY motif is important to the structure of mouse, zebrafish or chicken CXCR4 and what implications this would have on primordial germ cell migration. The importance of the arginine to both the function [27] and structure of Tre1 could also mean that the arginine of the DRY motif in CXCR4 is important for its structure and function.

Primordial germ cell migration is an important process to study as it serves as a model for cell migration. In many animals, the primordial germ cells are formed at a place distant to the presumptive gonads requiring the primordial germ cells to migrate to their target tissues. In order for the primordial germ cells to properly migrate to the presumptive gonads, the primordial germ cells are required to initiate migration, migrate through various tissues, evade or suppress cell death mechanisms and respond to directional cues. The study of primordial germ cell migration as a model for cell migration will help to better understand the mechanisms of cell movements, enabling the development of new techniques or approaches to treat cancer or other diseased states caused by improper cell migration.

## Methods

### Protein structure predictions

Protein structure predictions were generated for Tre1^+^ (GenBank ID: AAF46059) and Tre1^sctt^ using GPCR-ModSim [20] and GPCR-ITASSER [21]. Using squid rhodopsin (PDB ID: 2Z73) as a template, ten homology models were generated for both Tre1^+^ and Tre1^sctt^ by GPCR-ModSim. The best model (as judged by the lowest DOPE-HR score [47]) was chosen for further refinement of the loop regions. The DOPE-HR score is used to assess the quality of the models generated by examining the energy of the protein models. Five models were generated for each protein after loop refinement and again the best model was chosen based on DOPE-HR score.

Five models of both Tre1^+^ and Tre1^sctt^ were generated by GPCR-ITASSER and the best model as judged by the confidence score (C-score) was chosen for this study. C-score is an estimation of the structural prediction and is based on the threading alignments from LOMETS and convergence during the structural refinements [11].

### Building a system to reflect a Drosophila cellular membrane environment

The protein structure predictions (mtre1, msctt, gtre1 and gsctt) were embedded in a solvated (0.15 M NaCl) and pre-equilibrated POPE lipid bilayer using the Membrane Builder in the CHARMM-GUI [49,50]. For the mtre1 system, 101 Na^+^ and 112 Cl^-^ ions were added to neutralize the system and the system contained 37,557 water molecules. The upper and lower leaflets of the membrane contained 141 and 137 POPE lipids, respectively. The mtre1 system had a total of 153,870 atoms. The msctt system contained 110 Na^+^ and 120 Cl^-^ ions, 40,839 water molecules, 141 and 137 POPE lipids on upper and lower leaflets of the membrane, respectively, and 163,593 total atoms. The gtre1 system contained 69 Na^+^ and 80 Cl^-^ ions, 26,139 water molecules, 140 and 137 POPE lipids on upper and lower leaflets of the membrane, respectively, and 119,427 total atoms. The gsctt system contained 68 Na^+^ and 78 Cl^-^ ions, 26,102 water molecules, 139 and 140 POPE lipids on upper and lower leaflets of the membrane, respectively, and 119,423 total atoms.

### Molecular dynamics simulations

MD simulations were performed using the NAMD 2.8 simulation package [45]. The CHARMM22 [64,65] and CHARMM36 [66] force fields were used for proteins and lipids, respectively, and water molecules were described using TIP3P [67]. All systems were simulated at 310 K. Temperature and pressure were held constant with Langevin dynamics [45] and the Nose-Hoover Langevin piston [68,69]. Particle-mesh Ewald was used to calculate electrostatic interactions [70] and a 12 Å cut-off for van der Waals interactions was used. Each system was simulated on three compute nodes, each containing one Intel(R) Xeon(R) X5650 CPU (6 cores at 2.67 GHz), two Nvidia C2070 graphical processing units (GPUs) and 24 GB of RAM connected by QDR QLogic Infiniband.

After building the systems with the Membrane Builder in the CHARMM-GUI, six short (25 or 100 ps) equilibrium simulations were performed to gradually equilibrate the systems. Details for the equilibrium simulations can be found in reference [49]. Briefly, positional harmonic restraints were used on the protein backbone, protein side chains and ions. Additional harmonic restraints were used on the water molecules, to prevent water molecules from entering the hydrophobic region of the membrane, and the lipid head groups, to keep the lipid head groups level with the Z-axis. The restraints were reduced at each subsequent equilibrium simulation. The first two simulations used the NVT (constant volume and temperature) ensemble and the last four equilibrium simulations used the NPAT (constant pressure, area and temperature) ensemble. A timestep of 1 fs was used for the first three equilibrium simulations, which were 25 ps each. The last three equilibrium simulations used a 2 fs timestep and were run for 100 ps each [49]. Production runs began after the systems were equilibrated and used an NPT (constant pressure and temperature) ensemble and a 2 fs timestep. Harmonic restraints were not used in the production runs. Production runs were 262 ns, 258 ns, 270 ns, and 276 ns for mtre1, msctt, gtre1 and gsctt, respectively.

### Data analysis

Visual Molecular Dynamics 1.9 (VMD) [71] was used to visualize the trajectories and to perform the all-to-all RMSD calculations and the salt bridge analysis. The Voronoi Tesselation and Monte Carlo (VTMC) integration method was used to calculate the surface area per lipid in all model systems [52] to ensure the systems maintained a biologically relevant, fluid phase lipid bilayer. The Dictionary of Secondary Structure in Proteins [56,57] with the do_dssp interface supplied by GROMACS [72] was used to calculate the evolution of secondary structures over time for each model.

### Amino acid numbering

Amino acid residues are labeled using the single-letter code for the amino acid followed by the absolute sequence number. For example, arginine 134 is labeled R134. Tre1^sctt^ is missing eight amino acids compared to Tre1^+^; however, the absolute sequence number of amino acid residues studied in this protein is still used. For example, an aspartic acid residue in TM6 is labeled D266 in Tre1^+^ and D258 in Tre1^sctt^.

### Statistical analyses

Like any set of data, MD simulations are prone to statistical errors. The errors can be from inaccuracies in the model or inadequate sampling. For this reason, it is important to report the statistical uncertainty of values determined from simulations. In order to calculate the statistical uncertainty of different values in a simulation, the number of independent samples within a single simulation needs to be known. It has been suggested that estimation of a value of interest based on less than 20 statistically independent samples is considered unreliable [54]. To calculate the number of independent samples within a simulation, the decorrelation time must be calculated.

To calculate the decorrelation time, this study used the structural histogram analysis and automated effective sample size methods developed by the Zuckerman lab [73,74], as well as the block covariance overlap method (BCOM) from the Grossfield lab [53]. The effective sample size gives the degrees to which a simulation has sampled the conformational space of the protein and BCOM is a method used to measure the extent of convergence of a simulation. All of these tools are available through the LOOS (Lightweight Object-Oriented Structure) analysis library [75]. Only transmembrane Cα atoms were used in the decorrelation time calculations.

The decorrelation times as estimated by the structural histogram analysis and by the automated effective sample size are shown in Additional file 4: Table S1 and the decorrelation times calculated using the BCOM are shown in Additional file 5: Table S2. The results from the structural histogram analysis, the automated effective sample size calculation and the blocked covariance overlap method indicate that the systems have insufficient sampling and have not converged. This means that statistics generated from the data are not sufficient to draw statistically meaningful conclusions. This is not surprising. Microsecond simulations (or longer) with other GPCRs did not show convergence using these same methods [53]. Since the systems in this study have not converged, the values presented in this study represent a more qualitative assessment of the simulations.

## Abbreviations

GPCR: G protein-coupled receptor; Tre1: Trapped in endoderm-1; TM: Transmembrane; MD: Molecular dynamics; POPE: 1-palmitoyl-2-oleoyl-*sn*-glycero-3-phosphoethanolamine; GPCR-ITASSER: GPCR-Iterative Threading ASSEmbly Refinement; VTMC: Voronoi Tesselation and Monte Carlo integration method; RMSD: Root mean squared deviation; RMSF: Root mean squared fluctuation.

## Competing interests

The authors declare that they have no competing interests.

## Authors’ contributions

MMP, MHL, and CRC conceived the study. MMP performed the simulations and data analysis. MMP, MHL, and CRC wrote the manuscript. All authors read and approved the final manuscript.

## Supplementary Material

Additional file 1: Figure S1 RMSD of entire protein structures shows equilibration began at 150 ns. Description: Root mean squared deviation (RMSD) was calculated for each complete protein and is plotted over simulation time. The curves show the protein structures began to equilibrate after 150 ns. Click here for file

Additional file 2: Figure S2 Evolution of protein secondary structure over time. Description: The secondary structure of the proteins in each of the model systems was calculated and plotted over the simulation time with the do_dssp interface supplied by GROMACS [72]. Residues with the same secondary structure are in the same color. These plots show that the transmembrane regions of the proteins (blue) remain stable throughout the simulations. Click here for file

Additional file 3: Figure S3 Distances between Cα residues of TM3 and TM6 are similar in all model systems. Description: The distances between the Cα residues of R134 and D266 in Tre1^+^ and R135 and D258 in Tre1^sctt^ were calculated and plotted over simulation time. Click here for file

Additional file 4: Table S1 Approximate decorrelation times for the four different model systems. Description: τ_d_1 is the decorrelation time as estimated from the plot of σ^2^ (t) with step sizes 2, 4 and 5 [73]. τ_d_2 is the decorrelation time from the automated effective sample size calculation [74]. Both calculations are part of the LOOS analysis library [75]. ^a^ 10 bins were used, ^b^ 20 bins were used. Click here for file

Additional file 5: Table S2 Assessing convergence of the different model systems using the blocked covariance overlap method. Description: BCOM is the blocked covariance overlap method and BBCOM is the bootstrapped blocked covariance overlap. *t*_*1*_ - *t*_*3*_ are decorrelation times from fitting the BCOM/BBCOM curve to: *f(t) = k*_*1*_*e*^*-t/t1*^ *+ k*_*2*_*e*^*-t/t2*^ *+ k*_*3*_*e*^*-t/t3*^ *+ 1*[53]. The BCOM/BBCOM ratio decays to a final ratio of greater than 1 for each model system. This suggests that the systems have not yet converged. BCOM/BBCOM is part of the LOOS analysis library [75]. Click here for file
