# Supplementary figures and images for: Molecular dynamics simulations on the Tre1 G protein-coupled receptor: exploring the role of the arginine of the NRY motif in Tre1 structure
Source: BMC Struct Biol. 2013 Sep 18;13:15. doi: 10.1186/1472-6807-13-15 (PMC3848830; doi:10.1186/1472-6807-13-15)

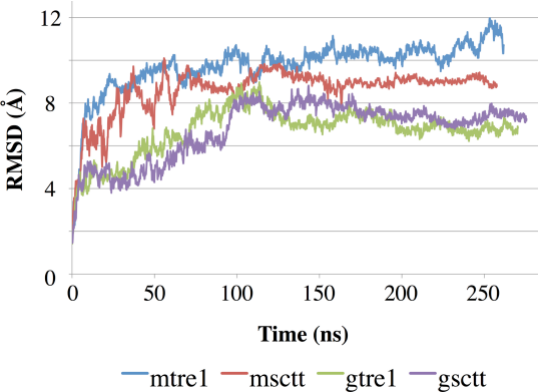

Supplement: Additional file 1: Figure S1 — RMSD of entire protein structures shows equilibration began at 150 ns. Description: Root mean squared deviation (RMSD) was calculated for each complete protein and is plotted over simulation time. The curves show the protein structures began to equilibrate after 150 ns. [file 1472-6807-13-15-S1.pdf]

**mtrel**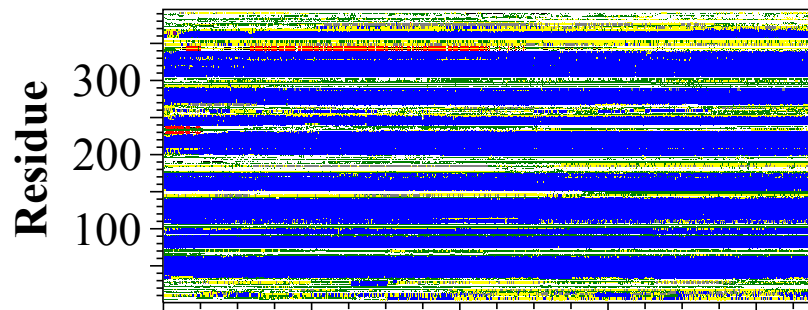**msctt**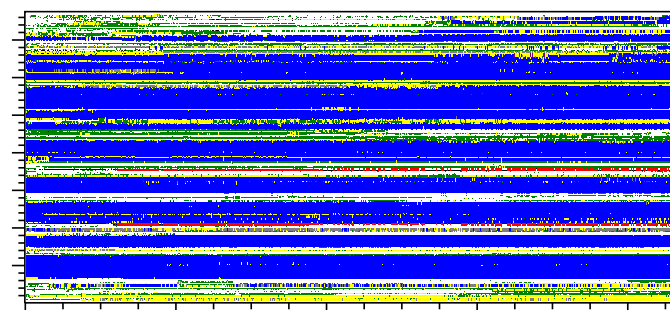**gtrel**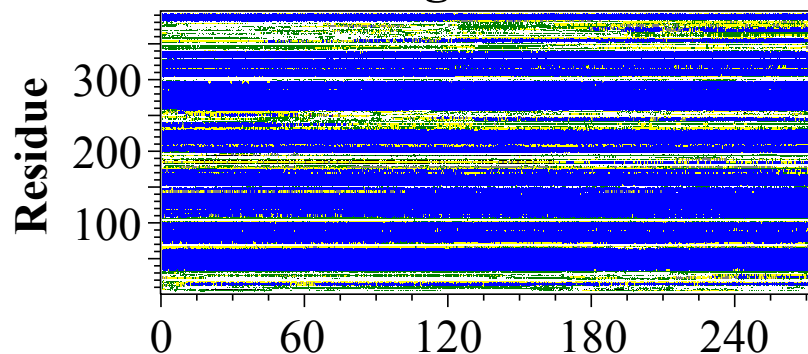**gsctt**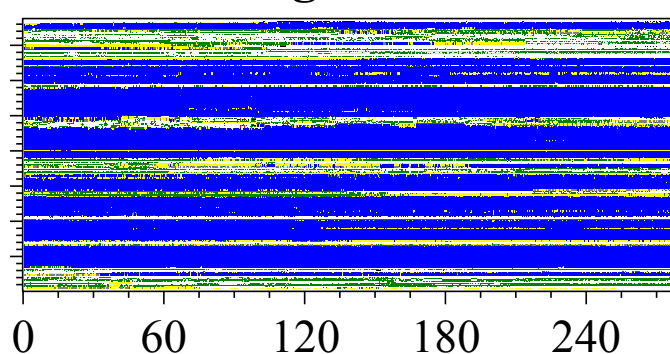

Coil B-Sheet B-Bridge Bend Turn A-Helix 5-Helix 3-Helix

Supplement: Additional file 2: Figure S2 — Evolution of protein secondary structure over time. Description: The secondary structure of the proteins in each of the model systems was calculated and plotted over the simulation time with the do_dssp interface supplied by GROMACS [72]. Residues with the same secondary structure are in the same color. These plots show that the transmembrane regions of the proteins (blue) remain stable throughout the simulations. [file 1472-6807-13-15-S2.pdf]

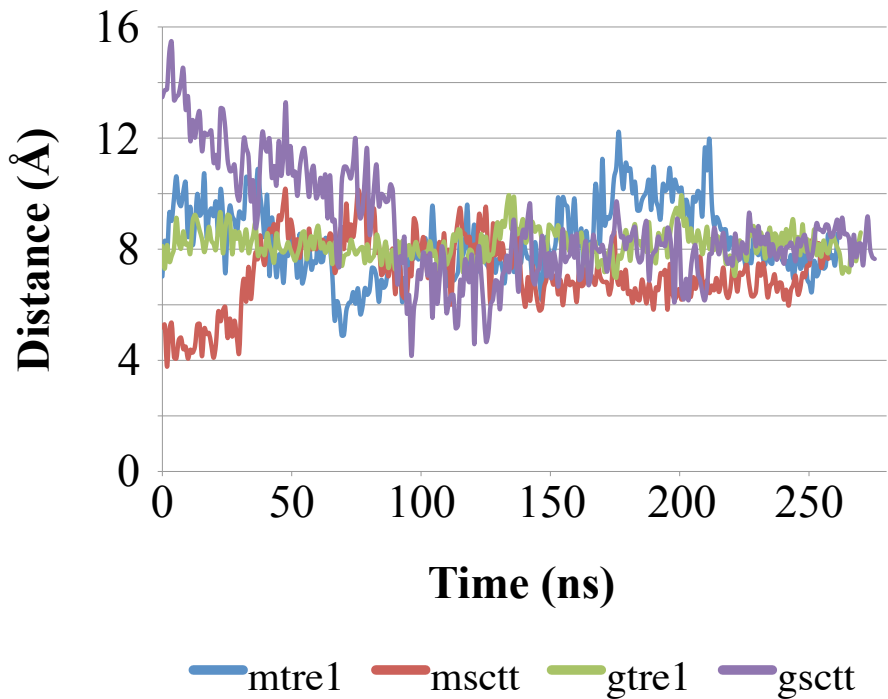

Supplement: Additional file 3: Figure S3 — Distances between Cα residues of TM3 and TM6 are similar in all model systems. Description: The distances between the Cα residues of R134 and D266 in Tre1+ and R135 and D258 in Tre1sctt were calculated and plotted over simulation time. [file 1472-6807-13-15-S3.pdf]
