# Supplementary material for: Molecular dynamics simulations on the Tre1 G protein-coupled receptor: exploring the role of the arginine of the NRY motif in Tre1 structure
Source: BMC Struct Biol. 2013 Sep 18;13:15. doi: 10.1186/1472-6807-13-15 (PMC3848830; doi:10.1186/1472-6807-13-15)
Supplement: Additional file 4: Table S1 — Approximate decorrelation times for the four different model systems. Description: τd1 is the decorrelation time as estimated from the plot of σ2 (t) with step sizes 2, 4 and 5 [73]. τd2 is the decorrelation time from the automated effective sample size calculation [74]. Both calculations are part of the LOOS analysis library [75]. a 10 bins were used, b 20 bins were used. [file 1472-6807-13-15-S4.pdf]

Model System	τ_d1 [a] (ns)	τ_d1 [b] (ns)	τ_d2 [a] (ns)	τ_d2 [b] (ns)
mtre1	24.0 – 29.1	17.4 – 18.9	165.8 – 178.7	77.1 – 84.9
msctt	18.6 – 23.7	14.1 – 15.9	139.2 – 184.4	72.9 – 84.2
gtre1	21.3 – 27.9	18.3 – 19.8	142.7 – 180.5	73.3 – 87.9
gsctt	19.8 – 25.2	15.0 – 19.8	165.5 – 189.5	82.5 – 89.5
