# Supplementary material for: Molecular dynamics simulations on the Tre1 G protein-coupled receptor: exploring the role of the arginine of the NRY motif in Tre1 structure
Source: BMC Struct Biol. 2013 Sep 18;13:15. doi: 10.1186/1472-6807-13-15 (PMC3848830; doi:10.1186/1472-6807-13-15)
Supplement: Additional file 5: Table S2 — Assessing convergence of the different model systems using the blocked covariance overlap method. Description: BCOM is the blocked covariance overlap method and BBCOM is the bootstrapped blocked covariance overlap. t1 - t3 are decorrelation times from fitting the BCOM/BBCOM curve to: f(t) = k1e-t/t1 + k2e-t/t2 + k3e-t/t3 + 1[53]. The BCOM/BBCOM ratio decays to a final ratio of greater than 1 for each model system. This suggests that the systems have not yet converged. BCOM/BBCOM is part of the LOOS analysis library [75]. [file 1472-6807-13-15-S5.pdf]

Model System	k_1	t_1 (ns)	k_2	t_2 (ns)	k_3	t_3 (ns)	BCOM/BBCOM
mtre1	5.0	2.2	2.1	62.0	0.4	983.0	1.7
msctt	2.1	3.9	1.4	67.1	0.3	1438.1	1.5
gtre1	2.2	1.1	0.8	7.1	1.0	138.0	1.4
gsctt	4.8	1.8	1.8	42.1	0.7	2247.1	1.7
